# Supplementary material for: Two Korean Endemic Clematis Chloroplast Genomes: Inversion, Reposition, Expansion of the Inverted Repeat Region, Phylogenetic Analysis, and Nucleotide Substitution Rates
Source: Plants (Basel). 2021 Feb 19;10(2):397. doi: 10.3390/plants10020397 (PMC7922562; doi:10.3390/plants10020397)
Supplement: Supplementary file 1 [file plants-10-00397-s001.zip › Table S2.docx]

**Table S2.** Accession numbers for species included in the phylogenetic substitution rates analysis.

| **Family** | **Groups** | | **Species** | **Accession numbers** |
| --- | --- | --- | --- | --- |
| Ranunculaceae | Other Ranunculaceae | | *Hydrastis canadensis* | KY085918 |
|  | Other Ranunculaceae | | *Thalictrum coreanum* | NC_026103 |
|  | Other Ranunculaceae | | *Urophysa henryi* | NC_039744 |
|  | Other Ranunculaceae | | *Adonis coerulea* | MK253469 |
|  | Other Ranunculaceae | | *Megaleranthis saniculifola* | NC_012615 |
|  | Other Ranunculaceae | | *Trollius chienesis* | NC_031849 |
|  | Other Ranunculaceae | | *Caltha palustris* | MK253465 |
|  | Other Ranunculaceae | | *Gymnaconitum gymnandrum* | NC_033341 |
|  | Other Ranunculaceae | | *Aconitum angustius* | NC_036357 |
|  | Other Ranunculaceae | | *Aconitum reclinatum* | MF186593 |
|  | Other Ranunculaceae | | *Aconitum barbatum* | MK253470 |
|  | Other Ranunculaceae | | *Aconitum barbatum var. hispidum* | KT820664 |
|  | Other Ranunculaceae | | *Aconitum kusnezoffii* | MK253471 |
|  | Other Ranunculaceae | | *Aconitum austrokoreense* | KY407559 |
|  | Other Ranunculaceae | | *Aconitum episcopale* | NC_038096 |
|  | Other Ranunculaceae | | *Aconitum delavayi* | MG678802 |
|  | Other Ranunculaceae | | *Actaea dahurica* | MK253463 |
|  | Other Ranunculaceae | | *Calianthemum alatavicum* | MK253466 |
|  | Other Ranunculaceae | | *Halerpestes sarmentosa* | MK253457 |
|  | Other Ranunculaceae | | *Ranunculus repens* | NC_036976 |
|  | Other Ranunculaceae | | *Ranunculus macranthus* | NC_008796 |
|  | Anemoneae | | *Anemone trullifolia* | MH205608 |
|  | Anemoneae | | *Pulsatilla chinensis* | NC_039452 |
|  | Anemoneae | | *Anemoclema glaucifolium* | NC_037194 |
|  | Anemoneae | *Clematis* | *Clematis trichotoma* (CTR) | NC_043828 |
|  | Anemoneae | *Clematis* | *C. tangutica* (CTA) | MK253446 |
|  | Anemoneae | *Clematis* | *C. repens* (CRE) | NC_039578 |
|  | Anemoneae | *Clematis* | *C. uncinata* (CUN) | NC_039846 |
|  | Anemoneae | *Clematis* | *C. terniflora* (CTE) | NC_028000 |
|  | Anemoneae | *Clematis* | *C. brachyura* (CBR) | NC_042793 |
|  | Anemoneae | *Clematis* | *C. heracleifolia* (CHE) | NC_039845 |
|  | Anemoneae | *Clematis* | *C. brevicaudata* (CBRE) | NC_039579 |
|  | Anemoneae | *Clematis* | *C. aethusifolia* (CAE) | NC_039577 |
|  | Anemoneae | *Clematis* | *C. flabellate* (CFU) | KM652489 |
|  | Anemoneae | *Clematis* | *C. macropetala* (CMA) | NC_041477 |
|  | Anemoneae | *Clematis* | *C. alternata* (CLA) | NC_039577 |
