# Supplementary material for: Two Korean Endemic Clematis Chloroplast Genomes: Inversion, Reposition, Expansion of the Inverted Repeat Region, Phylogenetic Analysis, and Nucleotide Substitution Rates
Source: Plants (Basel). 2021 Feb 19;10(2):397. doi: 10.3390/plants10020397 (PMC7922562; doi:10.3390/plants10020397)
Supplement: Supplementary file 1 [file plants-10-00397-s001.zip › Table S1.docx]

| **Taxa** | **Length of genome (bp)** | | | | **Number of genes** | | | | | **Pseudogenes** | **GC content (frequency/%)** | | | |
| --- | --- | --- | --- | --- | --- | --- | --- | --- | --- | --- | --- | --- | --- | --- |
|  | **Genome size** | **LSC** | **SSC** | **IR** | | **Total** | **CDS** | **tRNAs** | **rRNAs** |  | **Total** | **LSC** | **SSC** | **IR** |
| *C. acerifolia* | 159,552 | 79,315 | 18,145 | 31,046 | | 134 | 89 | 36 | 8 | 3(2 *infA*,*rpl32*) | 37.9 | 36.3 | 31.1 | 42 |
| *C. alternata* | 159,476 | 79,283 | 18,113 | 31,037  (31,043) | | 134 | 89 | 36 | 8 | 3(2 *infA*,*rpl32*) | 38 | 36.4 | 31.3 | 42 |
| ***C. brachyura*** | 159,532 | 79,342 | 18,107 | 31,042 | | 134 | 89 | 36 | 8 | 3(2 *infA*,*rpl32*) | 38 | 36.3 | 31.3 | 42.1 |
| *C. brevicaudata* | 159,583 | 79,339 | 18,112 | 31,066 | | 134 | 89 | 36 | 8 | 3(2 *infA*,*rpl32*) | 38 | 36.3 | 31.3 | 42 |
| *C. fusca* var. *coreana* | 159,609 | 79,480 | 18,053 | 31,038 | | 134 | 89 | 36 | 8 | 3(2 *infA*,*rpl32*) | 38 | 36.3 | 31.4 | 42 |
| *C. heracleifolia* | 159,565 | 79,323 | 18,110 | 31,066 | | 134 | 89 | 36 | 8 | 3(2 *infA*,*rpl32*) | 38 | 36.3 | 31.3 | 42 |
| *C. macropetala* | 159,647 | 79,442 | 18,129 | 31,038 | | 134 | 89 | 36 | 8 | 3(2 *infA*,*rpl32*) | 38 | 36.3 | 31.2 | 42 |
| *C. repens* | 159,507 | 79,156 | 18,268 | 30,967  (30,968) | | 134 | 89 | 36 | 8 | 3(2 *infA*,*rpl32*) | 38 | 36.3 | 31.2 | 42 |
| *C. tangutica* | 159,499 | 79,293 | 18,118 | 31,044 | | 134 | 89 | 36 | 8 | 3(2 *infA*,*rpl32*) | 37.9 | 36.3 | 31.3 | 42 |
| *C. terniflora* | 159,528 | 79,328 | 18,110 | 31,045 | | 134 | 89 | 36 | 8 | 3(2 *infA*,*rpl32*) | 38 | 36.3 | 31.4 | 42 |
| ***C. trichotoma*** | 159,170 | 79,089 | 17,997 | 31,042 | | 134 | 89 | 36 | 8 | 3(2 *infA*,*rpl32*) | 38 | 36.4 | 31.4 | 42 |
| *C. uncinata* | 159,524 | 79,349 | 18,099 | 31,038 | | 134 | 89 | 36 | 8 | 3(2 *infA*,*rpl32*) | 38 | 36.4 | 31.5 | 42 |

**Table S1.** Summary of the chloroplast genomes of *Clematis* species used in this study. LSC, large single copy; SSC, small single copy; IR, inverted repeat.
