# Supplementary figures and images for: Two Korean Endemic Clematis Chloroplast Genomes: Inversion, Reposition, Expansion of the Inverted Repeat Region, Phylogenetic Analysis, and Nucleotide Substitution Rates
Source: Plants (Basel). 2021 Feb 19;10(2):397. doi: 10.3390/plants10020397 (PMC7922562; doi:10.3390/plants10020397)

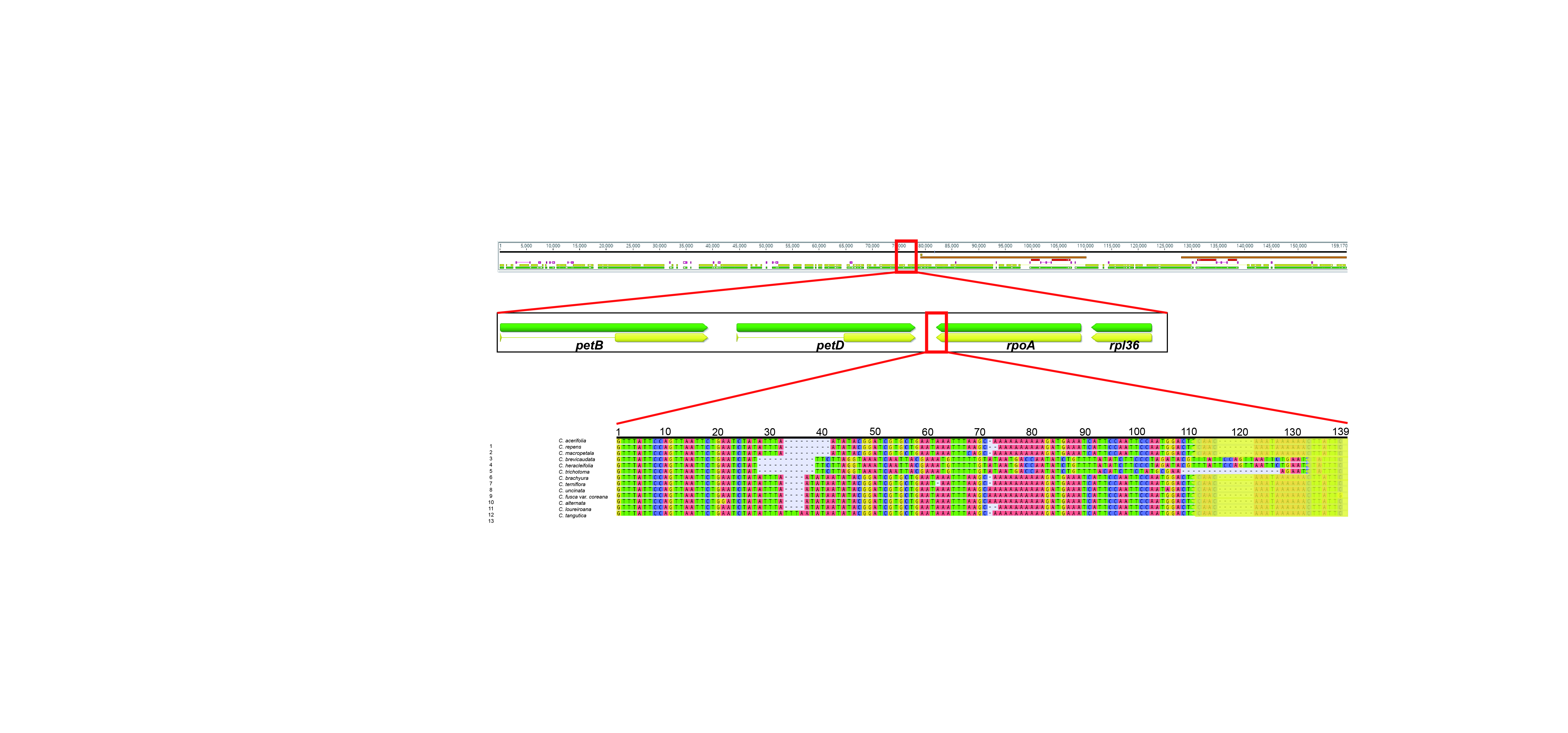

Supplement: Supplementary file 1 [file plants-10-00397-s001.zip › Figure S1.jpg]

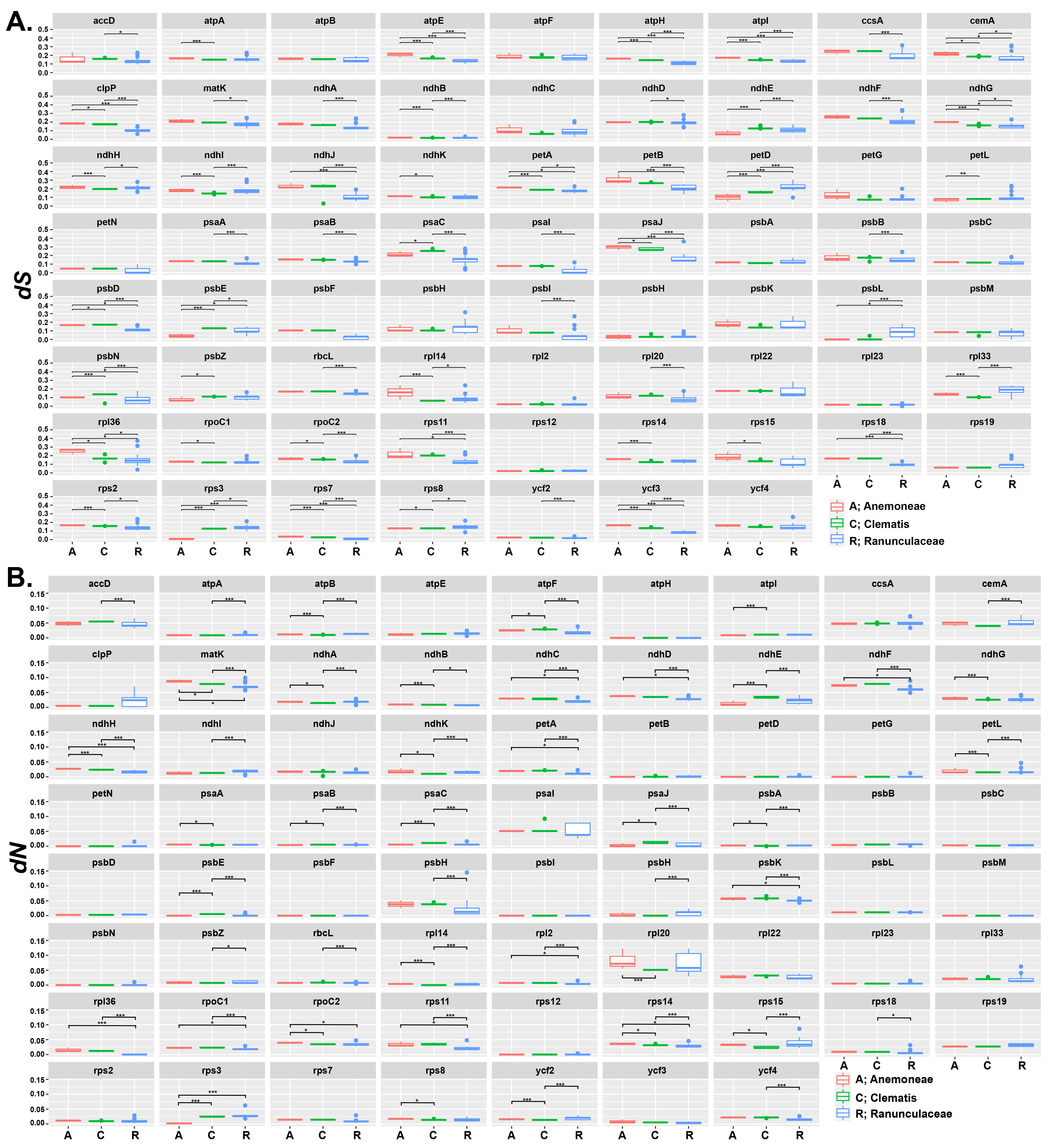

Supplement: Supplementary file 1 [file plants-10-00397-s001.zip › Figure S2.jpg]
